# The plasma exosomes from patients with primary Sjögren’s syndrome contain epithelial cell–derived proteins involved in ferroptosis

**DOI:** 10.1007/s00109-023-02361-0

**Published:** 2023-09-01

**Authors:** Xin Peng, Lei Hou, Xue Wu, Zhengqi Liu, Yun Wang, Ping Zeng, Ying Yang, Wukai Ma, Peng Yang

**Affiliations:** 1 Guizhou University of Traditional Chinese Medicine, Guiyang, 550002 China; 2 Department of Rheumatology and Immunology, Guizhou Provincial Traditional Chinese and Western Medicine Hospital, Guizhou University of Traditional Chinese Medicine, Guiyang, 550003 China

**Keywords:** Primary Sjogren’s syndrome, Exosomes, Proteomic, Ferroptosis

## Abstract

**Abstract:**

Primary Sjögren’s syndrome (pSS) is an autoimmune disease represented by exocrine gland epithelial cell lesions. However, the mechanism underlying these lesions remains unclear. This study analyzed the plasma exosomes of pSS patients using proteomics and revealed the presence of 24 differentially expressed proteins (DEPs) involved in the primary biological processes and signaling pathways related to ferroptosis. The DEPs enriched in the ferroptosis-related items were represented by downregulated ceruloplasmin (CP) and transferrin (TF). CC analysis of GO enrichment showed that CP and TF were localized at the apical plasma membrane, which is currently found only in epithelial cells. PPI analysis indicated that these exosomal DEPs formed a clustering network containing CP and TF. Among them, C5, C9, Haptoglobin (HP), and SERPING1 interacted directly with CP and TF. Notably, the expression of these proteins significantly decreased in both the pSS and secondary Sjögren’s syndrome (sSS) plasma exosomes but not in non-autoimmune sicca syndrome (nSS). In addition, their expression levels were significantly different in the exosomes and plasma. More importantly, the plasma and salivary exosomes of pSS patients contain higher levels of exocrine gland epithelial autoantigens SSA and SSB than those of healthy controls, and epithelial cells with positive labial glands biopsy (LGB) were more susceptible to ferroptosis than those with negative LGB. The results indicated that ferroptosis may be closely related to SS epithelial cell lesions.

**Key messages:**

• pSS plasma exosomes contain epithelial cell–derived proteins involved in ferroptosis.

• Complement C5 and C9 may be new molecules involved in ferroptosis and play a crucial role in pSS epithelial cell pathology.

• The serum exosomes from pSS patients, not nSS patients, contain ferroptosis-related proteins.

• The changes in the ferroptosis-related protein content in the exosomes can better reflect the state of the epithelial cell lesions than those in the plasma.

**Supplementary Information:**

The online version contains supplementary material available at 10.1007/s00109-023-02361-0.

## Introduction

Primary Sjögren’s syndrome (pSS) is a relatively prevalent chronic autoimmune disease marked by exocrine gland destruction and dysfunction and mainly involves the salivary and lacrimal glands. The main clinical manifestations are xerostomia and xeropthalmia but are often accompanied by musculoskeletal symptoms and systemic manifestations, significantly affecting the quality of life [1, 2]. The current treatment primarily follows a palliative strategy, focusing on exocrine fluid replacement or stimulating the secretion of the remaining exocrine glands [3, 4]. However, the existing treatments are mostly unsuccessful in improving symptoms and disease progression [4, 5]. Consequently, it is necessary to determine the underlying mechanism of exocrine gland injury from a new perspective to fully understand pSS pathogenesis and possibly reveal new targets for therapeutic intervention.

Previous studies have shown that the exocrine gland epithelial cells display many highly expressed apoptotic proteins Fas, FasL, and caspase-3 [6, 7], suggesting that apoptosis is closely related to epithelial cell lesions. However, no effective target has been found to treat epithelial injury. Ferroptosis is a novel type of iron-dependent programmed cell death distinct from apoptosis and closely related to GPX4 enzyme inactivation [8, 9]. In addition to inducing targeted cell death, it can activate the signaling pathway related to inflammation response [10, 11]. The characteristics of the subsequent cell death are highly consistent with the inflammatory response of exocrine gland lesions, suggesting the presence of a new death pathway in pSS epithelial cell damage. Since blood exosomes contain information molecules of originating lesions cells, which can reflect cytopathic changes to some extent [12–14], they have attracted increasing attention with regard to tumor pathogenesis [12], autoimmune disease [15], and cardiovascular disease [16]. Investigating blood-derived exosomes as targets can reveal the epithelial cell lesion mechanism in Sjögren’s syndrome (SS) patients.

Exosomes are small, lipid bilayer encapsulated vesicles with diameters of about 30–150 nm released by cells into the bodily fluids. Exosomes contain many information molecules, including proteins, RNA, DNA, and lipids that can reflect changes in the properties of the originating cells [13, 14]. Exosomes reportedly play an essential role in intercellular communication, both locally and systemically, as they transfer their contents between the recipient and originating cells via various physiological and pathological processes. Compared with plasma proteins, the lipid bilayer membrane protects exosomal proteins from plasma protease hydrolysis, causing them to stabilize, resulting in more cytopathy-labeled information substances, higher concentrations, and easier detection [12, 17, 18]. This can promote disease diagnosis and investigation of the pathological mechanism. This study screens the differentially expressed proteins (DEPs) in pSS patients using proteomics technology and determines whether they are related to ferroptosis. Protein–protein interaction (PPI) technology reveals several new proteins involved in ferroptosis. An enzyme-linked immunosorbent assay (ELISA) is employed to verify the level of expression of the DEPs related to ferroptosis in patients suffering from pSS, secondary Sjögren’s syndrome (sSS), and non-autoimmune sicca syndrome (nSS) and to compare the expression differences of these proteins in the exosomes and plasma. The study results highlight the role of ferroptosis in epithelial cell lesions and provide new insight into pSS pathogenesis.

## Methods and materials

### The patients and healthy participants

From March 2020 to June 2021 (three months), a total of 101 pSS patients were recruited from the Second Affiliated Hospital of Guizhou University of Traditional Chinese Medicine, of which only 86 patients were able to meet the requirements of this study, and the remaining 15 patients were excluded due to long-term drug use or complications such as tumors, hypertension, hyperglycemia, and anemia. In this study, 18 pSS patients were used for proteomics research, and 18 healthy individuals were selected as controls. Sixty-eight pSS patients were used for validation of the target protein of the screening, while 46 healthy individuals, 18 pSS patients with secondary rheumatoid arthritis (RA), 46 RA patients, and 16 nSS patients were selected as controls. In addition, the labial glands of 12 patients were collected for the analysis of ferroptosis in epithelial cells, and 6 pSS patients, 6 nSS patients, and 6 healthy individuals were collected to investigate the expression changes of epithelial cell markers SSA and SSB in saliva and plasma exosome. The sSS and pSS diagnosis was made according to the exclusion and inclusion standards set by the American-European Consensus Group for SS [19]. The 2010 ACR/EULAR classification criteria were used to diagnose RA [20, 21]. Patients with nSS did not meet the diagnostic criteria for SS and RA. The healthy individuals (*n* = 11) were recruited from outpatients requiring a general medical examination. All subjects have no liver injury, kidney injury, hepatitis B virus infection and other serious diseases. The baseline parameters of the clinical characteristics are shown in Table S1, and the parameters related to disease diagnosis are shown in Table S2. The Ethics Committee of the Second Affiliated Hospital of Guizhou University of Traditional Chinese Medicine approved the study. All participants signed written permission for their blood samples to be used for medical research before the examinations.

### Clinical parameter analysis

All clinical parameters were obtained from the clinical laboratory according to the standard operating procedure. The scoring criteria of RA DAS28 were determined according to the literature [20]. Schirmer’s I test was performed according to the standards presented in the available literature [19]; positive meant that the length of the moistened filter paper was less than 5 mm per min (≤ 5 mm/5 min). The unstimulated salivary flow was determined according to previous studies [19]; positive meant that the amount of saliva per 15 min was less than 1.5 mL (≤ 1.5 mL/15 min).

### Plasma samples

EDTA-treated venous blood was used to prepare the plasma samples of all the participants. These samples were subjected to centrifugation for 10 min and 30 min at 1000 g and 3000 g (4 °C), respectively, for cell and cell debris removal, after which the supernatant was stored at − 80 °C to perform the downstream analysis.

### Exosome isolation

The plasma exosomes were isolated using ultracentrifugation (UC) with some modifications [22]. Briefly, the plasma samples were thawed at 37 °C and subjected to centrifugation for 5 min and 20 min at 500 g and 2000 g, respectively, at 4 °C for cell and cell debris removal. This was followed by UC at 10,000 g at 4 °C for 45 min to eliminate the bigger vesicles. Next, the supernatant was filtered using a 0.45-µm pore filter and subjected to UC at 100,000 g at 4 °C for 75 min. The supernatant was then disposed of, followed by the resuspension of the exosome pellets in 200 µL PBS, 20 µL for electron microscopy, and 10 µL for particle size measurement. The remaining exosomes were stored at − 80 °C.

### Transmission electron microscopy (TEM)

The exosome samples (10 µL) were added dropwise onto 100-mesh formvar-coated copper grids for 1 min, followed by uranyl acetate (10 µL) for 1-min precipitation, while absorbing the floating liquid using a filter paper. After a 5–10 min room-temperature drying process, the TEM images were obtained at 80 kV.

The labial glandular tissue of 1 to 2 mm^3^ was fixed with 2.5% glutaraldehyde and osmium in turn. This was followed by dehydration, infiltration, embedding, and sectioning (80–100 nm). Finally, uranium–lead double staining and electron microscope observation was performed.

### Detection of surface markers of exosomes

The surface markers CD9 and TSG101 of exosomes were detected using Western Blot (WB). Briefly, 12% sodium dodecyl sulfate (SDS)-polyacrylamide gel was used to separate 20 µg protein extract; after which, a semi-dry transfer system was employed for relocation to a PVDF membrane. Next, 5% evaporated skimmed milk containing TBS-Tween 20 (0.05%) was used for blocking at room temperature for 2 h, followed by an overnight incubation at 4 °C on a membrane with primary antibodies against CD9 (ab92726, Abcam, Cambridge, UK) and TSG101 (ab125011, Abcam, Cambridge, UK). Next, the membranes were incubated for 2 h with HRP-coupled secondary antibodies (Feiyi Biotech, Wuhan, China). Photographic film and ECL blotting detection reagents were employed for protein band visualization (Thermo, Waltham, MA, USA).

The exosomal surface marker CD63 was analyzed by flow cytometry (FCM) analysis with vesicles pre-absorbed on latex beads (4% polystyrene latex beads, ThermoFisher, USA). The bead-exosome aggregates were labeled with the fluorescence-labeled antibodies anti-CD63 (1:200 eBioscience).

### Nanoparticle tracking analysis (NTA)

The frozen exosome samples were thawed in 25 °C water and diluted with 1 × PBS using a Nano Gold system (Izon Science Ltd, Christchurch, New Zealand).

### Protein concentration assay

The exosomal protein concentration was determined using a BCA protein assay kit (Boster Biological Technology, Wuhan, China) following the instructions of the manufacturer. A multifunctional enzyme labeling instrument (Varioskan LUX, Thermo Fisher Scientific, USA) was used to determine the total exosomal proteins.

### Label-free quantitative proteomic analysis

#### Plasma sample grouping

Eighteen patients with pSS were randomly divided into three groups, each containing the mixed plasma of six patients, labeled pSS1-6, pSS7-12, and pSS13-18, respectively. Moreover, eighteen healthy individuals were randomly divided into three groups via the same method and labeled HC1-6, HC7-12, and HC13-18.

#### Exosomal protein preparation

Briefly, the plasma samples of three pSS patients and three healthy controls were subjected to a 10-min centrifugation process at 1000 g, followed by a 15-min centrifugation at 17,000 g and 4 °C, and UC for 1 h at 4 °C and 200,000 g. Then, the supernatant was discarded, followed by 1 × PBS resuspended precipitation. Urea particles were added until a concentration of 8 M was reached and shaken until fully dissolved. Ultrasonic crushing on ice, 30% energy, ultrasonic for 1 s, stop for 1 s, was done cumulatively for 2 min. Centrifuge 14,000 g for 20 min, collect supernatant, 10 µL for protein quantification using the BCA protein assay kit, and freeze the rest at − 80 °C [22].

#### SDS-PAGE separation

Here, 5 × loading buffer was added to10-µg protein aliquots taken from the respective samples, followed by a 5-min boiling process. Next, 11.5% SDS-PAGE gel was used to separate the proteins at a constant current of 15 mA for 80 min, followed by Coomassie Blue R-250 staining to visualize the protein bands.

#### Protein digestion and desalination

A 100-µg aliquot of extracted proteins from each sample was subjected to a reduction process, followed by the addition of 200 mM dithiothreitol (DTT) solution and incubation at 37 °C for 1 h. The samples were diluted four times by adding 25 mM ammonium bicarbonate (ABC) buffer, followed by the addition of trypsin (trypsin: protein = 1:50), overnight incubation at 37 °C, and a 50-µL 0.1% FA addition to terminate the digestion process. Then, 100 µL of 100% ACN was used to wash the C18 column; after which, the sample was centrifuged for 3 min at 1200 rpm. The column was rinsed once using 100 µL of 0.1% FA and subjected to a 3-min centrifugation at 1200 rpm. The sample was added after replacing the EP tube and centrifuged for 3 min at 1200 rpm. The column was rinsed twice using 100 µL of 0.1% FA, followed by centrifugation for 3 min at 1200 rpm and washing with 100 µL water. The EP tube was replaced, followed by elution with 70% ACN. The sample eluents were combined, lyophilized, and stored at − 80 °C until loading [23].

#### LC–MS/MS analysis

The solutions for mobile phases A and B were prepared, containing 0.1% formic acid and 100% water and 0.1% formic acid and 100% acetonitrile, respectively. Next, 10 µL of the mobile phase A solution was used to dissolve the preserved powder, which was subjected to centrifugation for 20 min at 14,000 g and 4 °C. Then, 1 µg of the supernatant was added to a C18 column. Linear gradient elution was employed to separate the peptides in an analytical column, which were assessed using a Q Exactive HF-X mass spectrometer (Thermo, Waltham, MA, USA) equipped with a Nanospray Flex™ (ESI) ion source. The spray voltage was 2.4 kV, and the ion transport capillary temperature was 275 °C, using a 3 × 10^6^ automatic gain control (AGC) target value, a scan range of m/z 350 to 1500, a resolution of 120,000 (at m/z 200), and an 80-s maximum ion injection time. Higher-energy collisional dissociation (HCD) was utilized to select and fragment the top 40 precursors of the full scan maximum abundance. The samples were evaluated via MS/MS at 27% normalized collision energy, 15,000 resolution (at m/z 200), a 45-s maximum ion injection time, and a 5 × 10^4^ AGC target value [24].

#### Data analysis

The raw MS information was analyzed using the Proteome Discoverer 2.4 software and searched against the *Homo sapiens* database in the Universal Protein Resource Knowledge Base (UniProtKB).The initial search included a 15-ppm precursor mass window according to the trypsin enzymatic cleavage rule. A mass tolerance of 20 ppm for the ion fragments and a limit of two missed cleavage sites were permitted. Methionine oxidation and N-terminal acetylation were deemed variable alterations during the database search, while cysteine carbamido methylation was seen as a fixed alteration. Furthermore, *P* < 0.01 was considered the limit for protein determination and the global false discovery rate (FDR) in the peptide-spectrum match (PSM). The proteins were considered accurately detected and present when indicated by a minimum of two different peptides and in at least two samples. These samples were submitted for further assessment [25].

#### Protein quantification and data processing

Each detected protein was subjected to label-free quantification (LFQ) by evaluating the intensity of the peptide signals. The protein abundance of the detected peptides was quantified via MaxLFQ algorithm activation in MaxQuant. The experiments utilized match-between-runs for the extraction of complete quantification data. In the case of low-abundance proteins presenting missing values, Perseus was used to provide a substitute value using the preselected parameters of the standard distribution. A two-way student’s *t*-test was employed to analyze the statistical significance and quantify the proteins. Proteins displaying *P* < 0.05 significance and a fold change (FC) of > 1.5 after comparing the two groups were designated as DEPs, while further assessment occurred using log2 (fold change).

### Bioinformatic analysis

During the Gene Ontology (GO) enrichment analysis, proteins were characterized via one of three categories, namely molecular function (MF), cellular compartment (CC), and biological process (BP) via GO annotation, according to the UniProt-GOA database (http://www.ebi.ac.uk/GOA/). The DEP abundance was compared to the identified proteins using Fisher’s exact test. Statistical significance was reflected by an amended *P*-value < 0.05. The Kyoto Encyclopedia of Genes and Genomes (KEGG) (http://www.genome.jp/KEGG/) database was utilized for enriched pathway identification via Fisher’s exact test to compare the detected proteins with the DEP abundance, while an adjusted *P*-value < 0.05 indicated statistical significance. The DEP pathways were clustered using Reactome pathway analysis (https://www.reactome.org/).

### ELISA

The concentrations of the human ceruloplasmin (CP), transferrin (TF), haptoglobin (HP), SERPING1, Ro (SSA), and La (SSB) were determined using ELISA kits supplied by EIAab Biological company (Wuhan, China) according to the instructions of the manufacturer.

### Detection of reactive oxygen species (ROS)

The main steps are as follows: Staining: after the frozen section is slightly dried, draw a circle around the tissue with a histochemical pen, drop the ROS dye in the circle, and incubate in a 37°-incubator away from the light for 30 min. DAPI counterstaining nuclei: put the slides in PBS (pH 7.4) and shake and wash them on the decolorization shaking table for 3 times, each time for 5 min. After the slices were slightly dried, DAPI dye was added in the circle, and incubated at room temperature in the dark for 10 min. Sealing slides: put the slides in PBS (pH 7.4), shake and wash them on the decolorization shaking table for 3 times, each time for 5 min. The slices were slightly dried and sealed with an anti-fluorescence quenching sealing agent. Finally, sections were observed under a fluorescence microscope and images were collected.

### Detection of total ferrum (Fe) and Fe^2+^ in labial gland tissue

The concentrations of total Fe and Fe^2+^ were determined using Colorimetry Assay Kits for total Fe and Fe^2+^ supplied by EIAab Biological company (Wuhan, China) according to the instructions of the manufacturer.

### Statistical analysis

The data were expressed as the median with the standard deviation of interquartile range. Mann–Whitney tests (unpaired samples) were employed to assess the variations between the two groups, and the statistical significance was reflected by *P* < 0.05. The GraphPad Prism V9 software (GraphPad Software, San Diego, CA, USA) was used for all the statistical evaluations.

## Results

### A total of 24 exosomal DEPs were identified in the plasm of the pSS patients

**Fig. 1:**
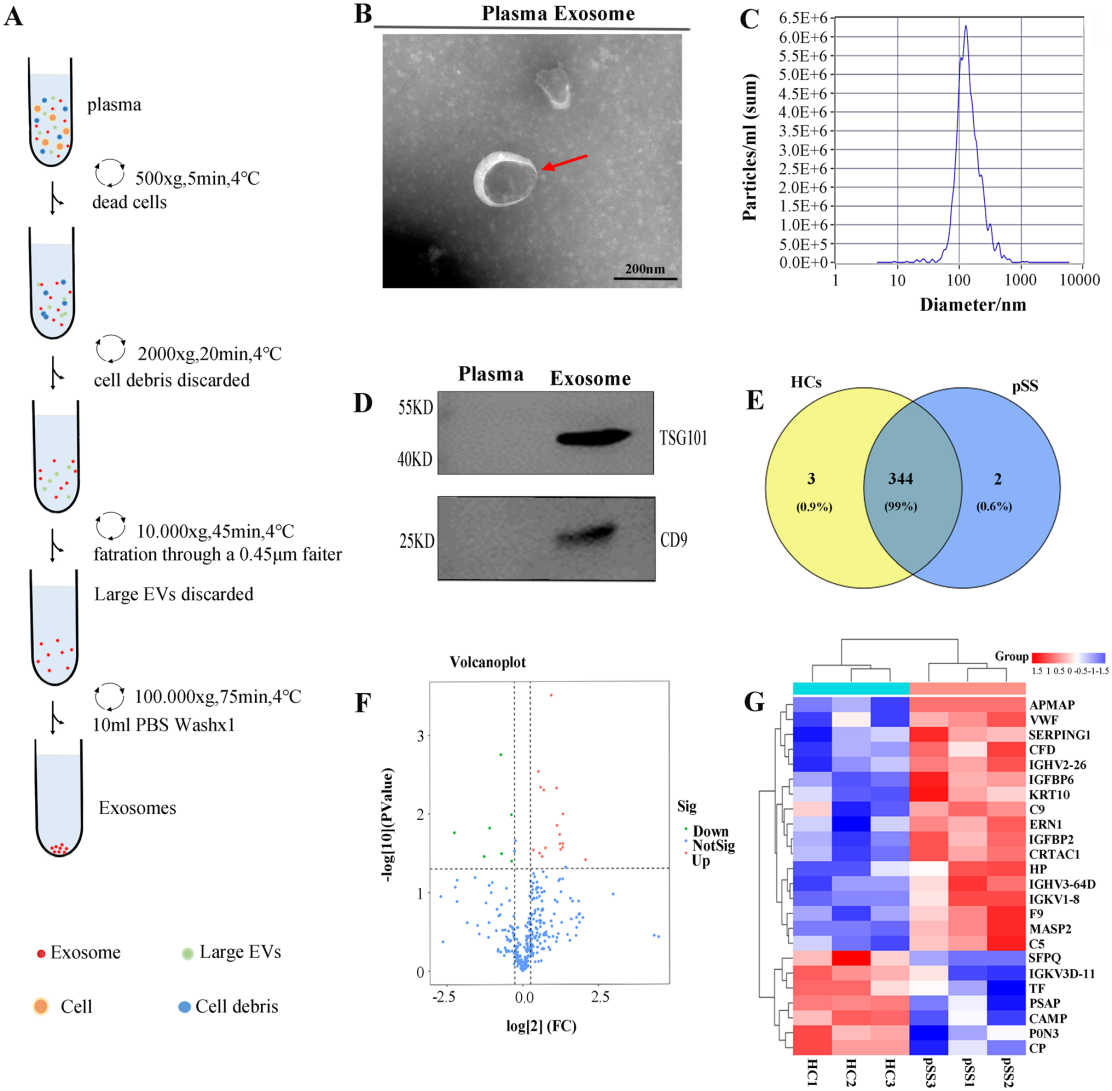
Identification and proteomic analysis of the plasma exosomes from the SS patients and control groups. **A** A flow chart of the plasma exosome separation via UC. **B** The TEM images of the mixed exosomes shown at 110,000 × magnification. Scale bar = 100 nm. **C** The plasma exosomal size dispersion obtained via NTA. **D** The WB assessment of the CD9 and TSG101 exosomal markers and plasma without exosomes as a control. **E** The Venn diagram showing the exosomal protein distribution. **F** A volcano map of the exosomal plasma DEPs. **G** A heat map showing the clustering of 24 DEPs. Red and green denote the respective high and low expression levels of the individual proteins

The flow chart of the isolated exosomes in the plasma samples via UC is shown in Fig. 1A. TEM showed typical cup-shaped vesicles (Fig. 1B), while NTA showed that these vesicles were approximately 131.8 nm in diameter (Fig. 1C). Moreover, no significant differences were evident between the total exosomal protein concentrations of the HC participants and pSS patients. The WB results indicated significant TSG101 and CD9 exosomal marker expression in the exosomal samples, but none in the plasma without exosomes (Fig. 1D). These findings demonstrated that the exosomes were successfully isolated in the plasma samples of the pSS and HC participants.

Label-free proteomics was used to detect 349 special proteins in the plasma exosomes, presenting 346 and 347 in the pSS and HC participants, respectively (Fig. 1E). A quantitative ratio exceeding 1.5 was considered upregulation, while a ratio below 0.667 was considered downregulation. Compared with the HCs, the pSS groups presented 24 DEPs, 17 of which were upregulated proteins, while seven were downregulated, as shown in the DEP volcano map (Fig. 1F) and clustering heat map (Fig. 1G).The detailed data regarding the protein downregulation and upregulation are shown in Tables 1 and 2.

**Table 1:** The downregulated DEPs between the plasma exosomes of pSS patients and HCs

Accession	Gene symbol	Protein name	FC	P
P23246	SFPQ	Splicing factor, proline- and glutamine-rich	0.7784	0.0402
A0A0A0MRZ8	IGKV3D-11	Immunoglobulin kappa variable 3D-11	0.7757	0.0103
P02787	TF	Serotransferrin	0.6166	0.0321
P07602	PSAP	Prosaposin	0.6107	0.0018
P49913	CAMP	Cathelicidin antimicrobial peptide	0.4712	0.0152
Q15166	PON3	Serum paraoxonase/lactonase 3	0.4154	0.0350
P00450	CP	Ceruloplasmin	0.2095	0.0173

**Table 2:** The upregulated DEPs between the plasma exosomes of pSS patients and HCs

Accession	Gene symbol	Protein name	FC	P
Q9HDC9	APMAP	Adipocyte plasma membrane-associated protein	4.2197	0.0388
P04275	VWF	von Willebrand factor	2.6864	0.0485
P05155	SERPING1	Plasma protease C1 inhibitor	2.5210	0.0101
P00746	CFD	Complement factor D	2.5180	0.0241
A0A0B4J1V2	IGHV2-26	Immunoglobulin heavy variable 2–26	2.5028	0.0268
P24592	IGFBP6	Insulin-like growth factor–binding protein 6	2.3883	0.0285
P13645	KRT10	Keratin, type I cytoskeletal 10	2.3567	0.0241
P02748	C9	Complement component C9	2.3401	0.0184
O75460	ERN1	Serine/threonine-protein kinase/endoribonuclease IRE1	2.2081	0.0141
P18065	IGFBP2	Insulin-like growth factor–binding protein 2	2.1802	0.0047
Q9NQ79	CRTAC1	Cartilage acidic protein 1	1.9371	0.0003
P00738	HP	Haptoglobin	1.6880	0.0273
A0A0J9YX35	IGHV3-64D	Immunoglobulin heavy variable 3-64D	1.6326	0.0050
A0A0C4DH67	IGKV1-8	Immunoglobulin kappa variable 1–8	1.5645	0.0351
P00740	F9	Coagulation factor IX	1.5143	0.0046
O00187	MASP2	Mannan-binding lectin serine protease 2	1.4740	0.0318
P01031	C5	Complement C5	1.4435	0.0029

### The main DEP functions and pathways are associated with ferroptosis

**Fig. 2:**
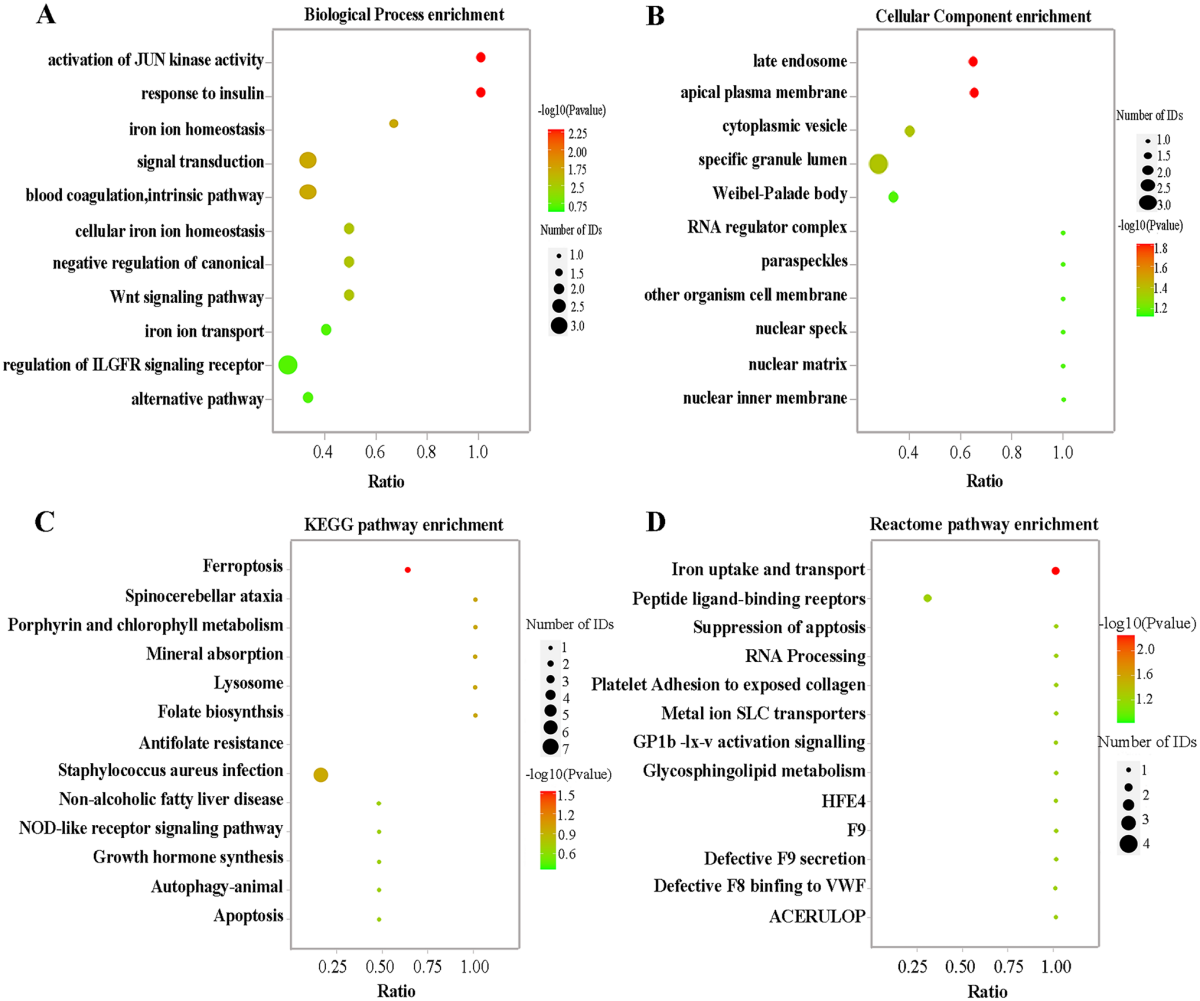
The biological information analysis of the exosomal DEPs. **A** Assessment of the BP enrichment. **B** Assessment of the CC enrichment. **C** Assessment of the KEGG pathway enrichment. **D** Assessment of the Reactome pathway enrichment

This study conducted a bioinformatics analysis of the main functions and pathways of the 24 DEPs to clarify whether the exosomal proteins in the plasma of the pSS patients were related to ferroptosis. The level of DEP enrichment was determined using Fisher’s exact test. The GO finding regarding the BP indicated the substantial involvement of the DEPs in JUN kinase activation (*P* = 0.0052), insulin response (*P* = 0.0052), iron ion homeostasis (*P* = 0.0148), and signal transduction (*P* = 0.0219). JUN kinase activity was associated with apoptosis, and iron ion homeostasis was related to ferroptosis, while the proteins enriched in these terms included CP, TF, and ERN1 (Fig. 2A and Table S3). GO analysis results for the CC section indicated that the DEPs were significantly enriched in the apical plasma membranes (*P* = 0.0154), late endosomes (*P* = 0.0154), and cytoplasmic vesicles (*P* = 0.0467), while the proteins enriched in these terms all contained TF (Fig. 2B and Table S4). KEGG assessment indicated complete DEP enrichment in the ferroptosis pathways (*P* = 0.029), while the corresponding proteins involved were CP and TF, as shown in Fig. 2C. Similarly, Reactome analysis showed primary DEPs enrichment during iron uptake and transport (*P* = 0.0056) (Fig. 2D), while the corresponding proteins involved were also CP and TF. The KEGG and Reactome pathway analysis results are shown in Tables S5 and S6, respectively. The functional enrichment analyses showed that the major biological functions of the DEPs were involved in the ferroptosis pathways.

### The new proteins involved in ferroptosis pathology were identified via PPI analysis

**Fig. 3:**
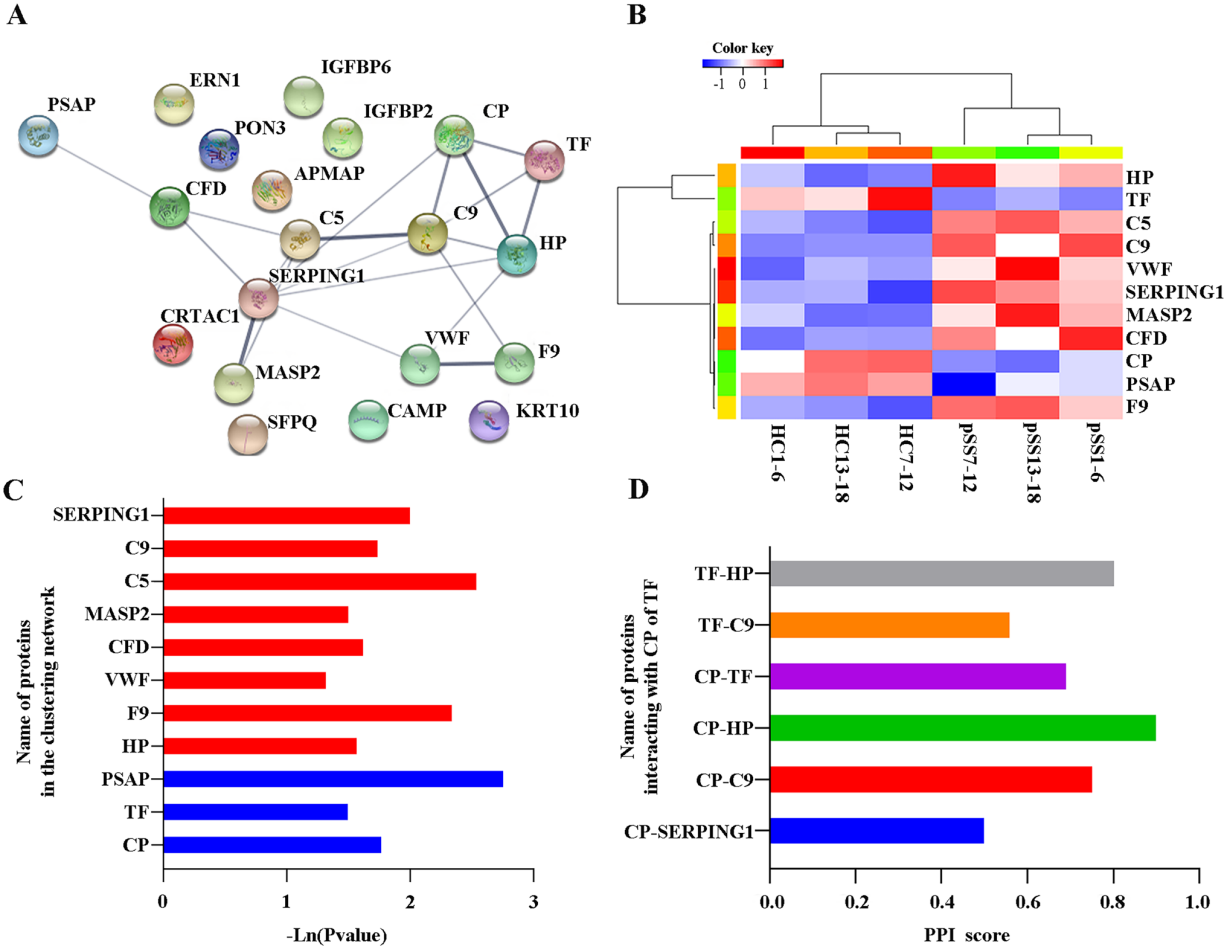
The PPI analysis of the exosomal DEPs. **A** The online STRING PPI network of 24 DEPs. The clustering network of the proteins related to CP and TF containing 11 nodes displaying 20 edges (at a clustering coefficient of 0.576 and a PPI enrichment *P*-value < 0.001). **B** The heatmap of the 11 proteins interacting with CP and TF. **C** The *P*-value of the relative expression levels of the proteins in the clustering network. Red indicates upregulation and blue indicates downregulation. **D** The PPI score of the proteins interacting with CP or TF

The STRING PPI database was used to determine the interaction between the target TF and CP proteins and other proteins to determine the differential proteins associated with the TF and CP involved in ferroptosis. The results showed that the clustering network participating in CP and TF displayed a total of 11 nodes exhibiting 20 edges (with a clustering coefficient of 0.576, and a PPI enrichment *P*-value < 1.0 E-16). The proteins directly interacting with the CP and TF included C5, C9, HP, and SERPING1 (Fig. 3A). It is suggested that these proteins may be involved in the pathological process of iron death, but this has not been confirmed yet. CP, TF, and PSAP protein expressions were significantly decreased in the CP and TF aggregation networks, while C5, C9, HP, and SERPING1 expressions displayed a substantial increase, compared with the healthy control (Fig. 3B and C). The proteins highly associated with CP or TF included C5, C9, HP, and SERPING1 (Fig. 3D).

### Validation of the DEPs associated with ferroptosis

**Fig. 4:**
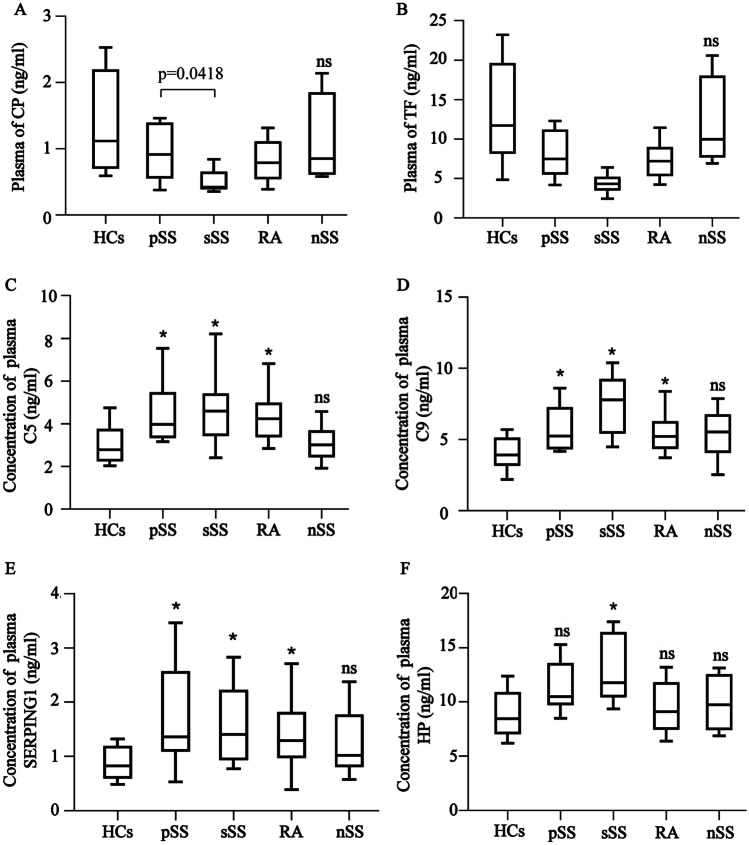
The expression levels of the proteins related to ferroptosis in the plasma exosomes. The observation objects include pSS, sSS, RA, and nSS. The ELISA test results are shown using a bar graph, shown as mean ± standard deviation. The pSS (*n* = 68), sSS (*n* = 18), RA (*n* = 46), and nSS (*n* = 16) groups were compared with the healthy group (*n* = 46) using a Mann–Whitney *U*-test (unpared).*P < 0.05, **P < 0.01, ns denotes the absence of statistically significant values. **A** The exosomal CP protein concentration. **B** The exosomal TF protein concentration. **C** The exosomal C5 protein concentration. **D** The exosomal C9 protein concentration. **E** The exosomal SERPING1 protein concentration. **F** The exosomal HP protein concentration

Combined with the GO, KEGG, Reactome, and PPI results, this study focuses on the expression levels of the exosomal proteins involved in ferroptosis, including CP, TF, C5, C9, HP, and SERPING1. Figure 4A shows that the expression of CP was substantially lower in pSS, sSS, and RA patients than in the HC group. Furthermore, its expression level was substantially lower in the sSS than in the pSS patients, while the nSS and HC groups displayed no substantial differences. The TF levels showed similar results. The expression of TF was considerably lower in the pSS, sSS, and RA groups than in the HC participants, while no significant changes were evident in then SS patients (Fig. 4B). The C5, C9, SERPING1, and HP content levels differed from those of CP and TF. Compared with the HC group, the C5 content in pSS, sSS, and RA groups increased substantially, and no significant variation was apparent in the SS participants, as shown in Fig. 4C. The C9 and SERPING1 content changes were similar to those in C5. The C9 detection results are shown in Fig. 4D, while the SERPING1 detection results are shown in Fig. 4E. However, the expression of HP in the pSS, nSS, and RA patients displayed no obvious changes from the HC group, except that it was considerably higher in the sSS group (Fig. 4F). These results suggested that these proteins were only related to the occurrence of the disease.

### The expression levels of the six DEPs associated with ferroptosis in the plasma

**Fig. 5:**
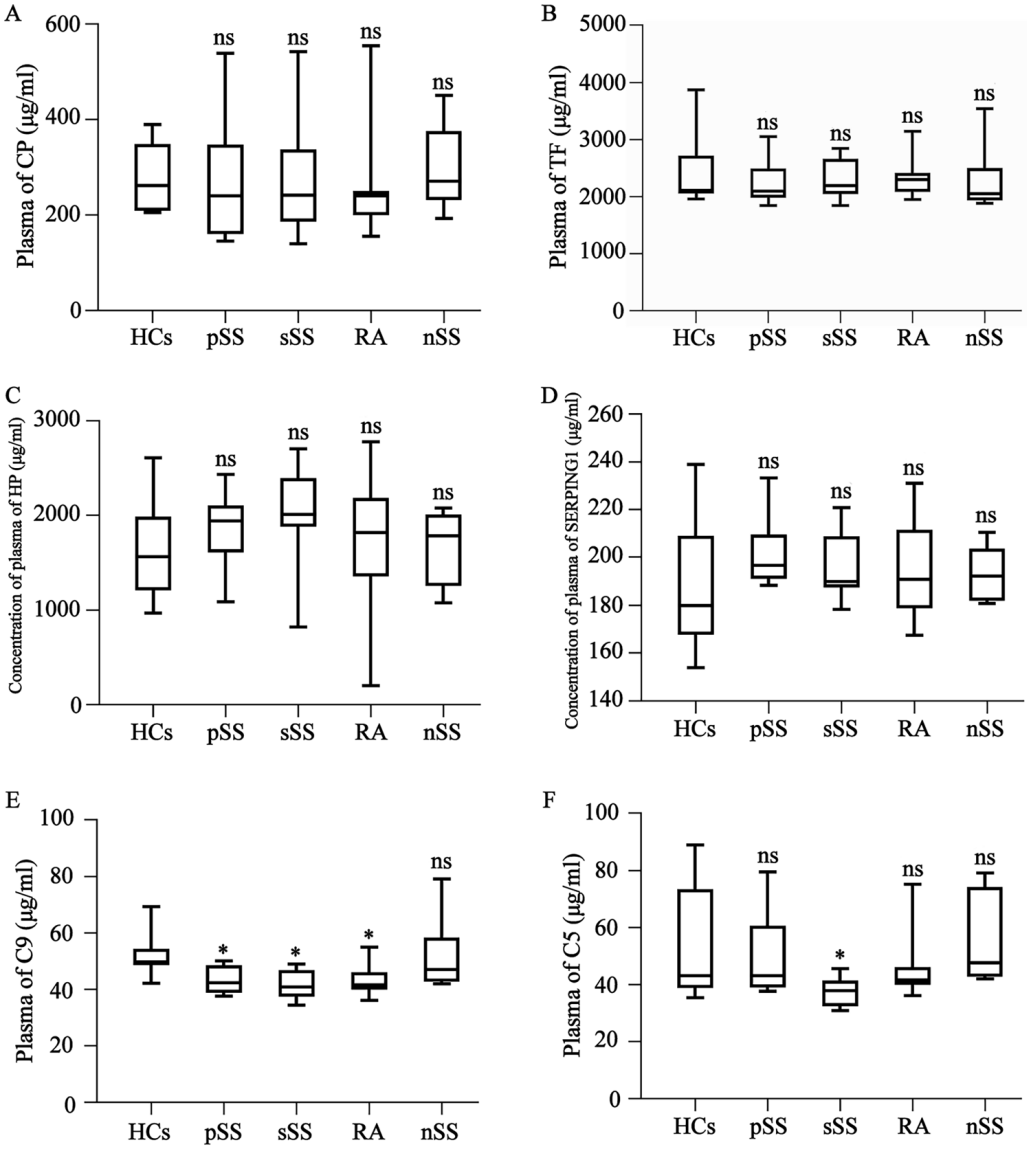
Expression levels of the proteins related to ferroptosis in the plasma of the patients. The observation objects include pSS (*n* = 68), sSS (*n* = 18), RA (*n* = 46), and nSS (*n* = 16). The test results are shown using a bar graph, shown as mean ± standard deviation. The disease groups were compared with the HCs via a Mann–Whitney *U*-test. *P < 0.05, ns denotes the absence of statistically significant values. The concentrations of **A** CP, **B** TF, **C** HP, **D** SERPING1, **E** C9, and **F** C5 in the plasma of the different groups

To further elucidate the role of the CP, TF, SERPING1, HP, C5, and C9 ferroptosis-related proteins in pSS pathogenesis, their expression levels in the plasma were determined, exhibiting differences from their exosomal expression levels. Compared with the HC group, the CP, TF, SERPING1, and HP expression levels in the pSS, sSS, RA, and nSS groups did not change significantly, while the detailed detection results are shown in Fig. 5A–D, respectively. However, the expression of C9 was substantially higher in the pSS, sSS, and RA patients, compared to the HC group, and no significant variation was evident in the nSS participants (Fig. 5E). Except for considerably high levels in the sSS patients, C5 expression displayed no significant changes in the other disease groups compared with the HC group (Fig. 5F).

### Abnormal expression of salivary gland epithelial cell markers SSA and SSB was found in pSS plasma and salivary exosomes

**Fig. 6:**
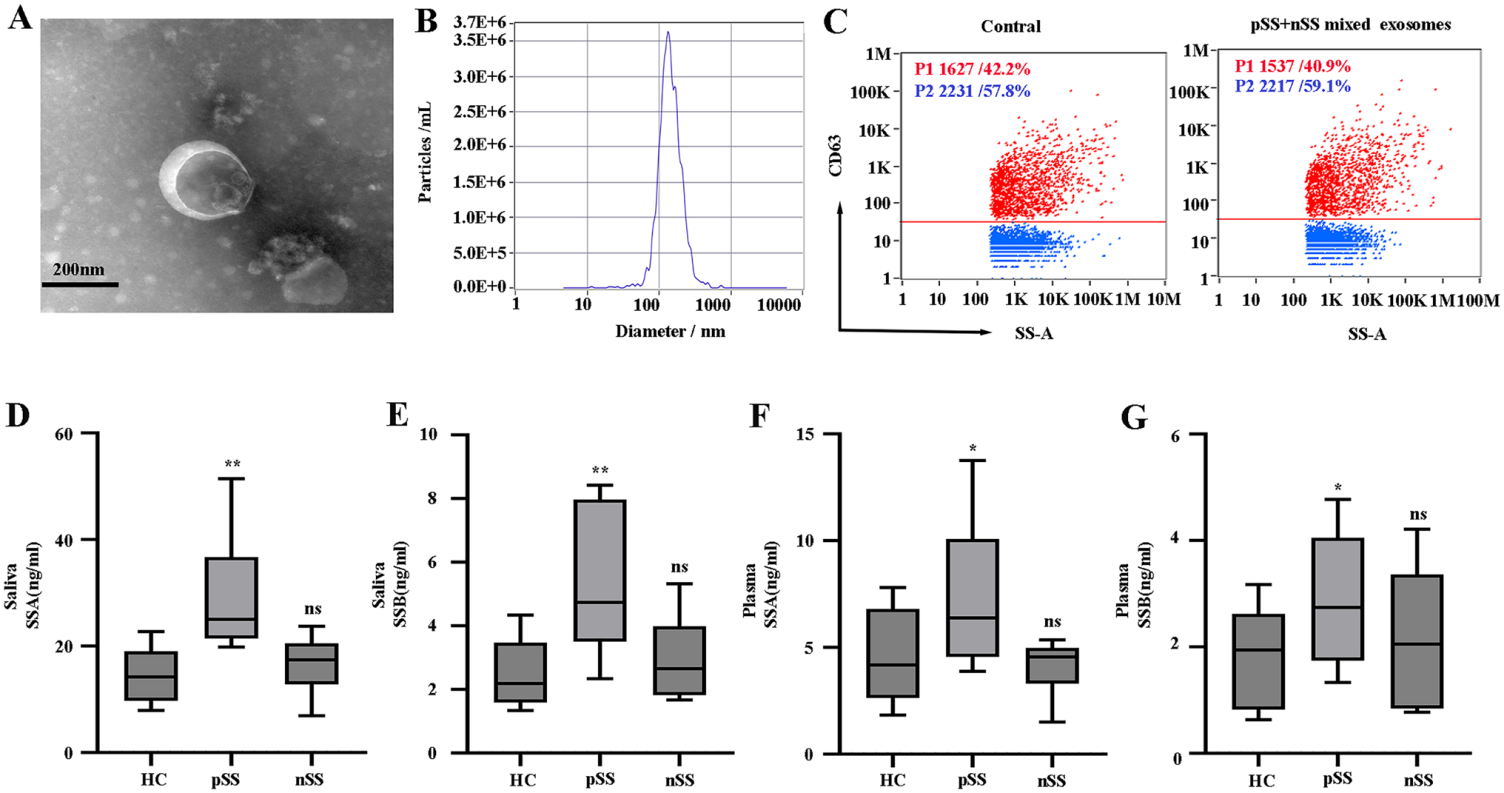
Detection of SSA and SSB markers related to salivary gland epithelial cells in exosomes. **A** The TEM images of the saliva exosomes are shown at 110,000 × magnification. Scale bar = 100 nm. **B** The salivary exosomal size dispersion was obtained using NTA. **C** The FCM assessment of the CD63 exosomal markers. **D** The salivary exosomal SSA protein concentration. **E** The salivary exosomal SSB protein concentration. **F** The plasma exosomal SSA protein concentration. **G** The plasma exosomal SSB protein concentration. The disease groups (*n* = 6) were compared with the HCs (*n* = 6) through a Mann–Whitney *U*-test. *P < 0.05, **P < 0.01

Autoantigenic salivary gland epithelial cell markers SSA and SSB were found in plasma and salivary exosomes to verify if the changes in plasma exosomal proteins CP and TF in pSS are related to the injury of salivary gland epithelial cells. TEM showed typical cup-shaped vesicles in saliva (Fig. 6A), while NTA showed that these vesicles were approximately 137.7 nm in diameter (Fig. 6B), and Flow Cytometry (FCM) results indicated significant CD63 exosomal marker expression in exosomal samples (Fig. 6C). Compared with the normal group, the contents of SSA and SSB in saliva exosomes of pSS were significantly higher than those of healthy controls (Fig. 6D and E). Similar results were also shown in plasma exosomes, with significantly higher levels of SSA and SSB in patients than in healthy controls (Fig. 6F and G), suggesting that abnormal plasma exosomal proteins may be associated with salivary gland epithelial cell injury.

### Ferroptosis was observed in labial glandular epithelial cells in pSS patients

**Fig. 7:**
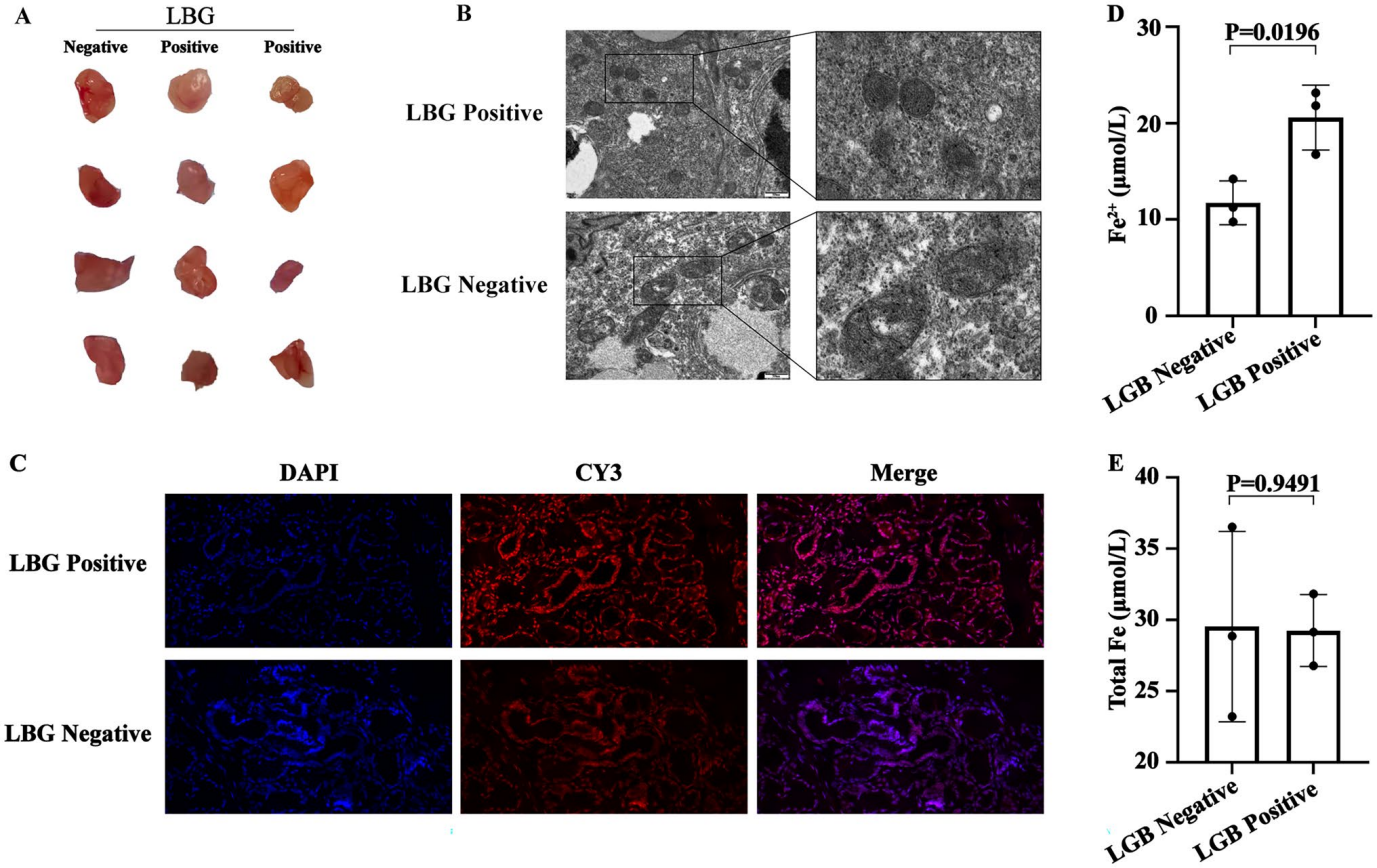
Detection of ferroptosis in labial gland tissue of pSS patients. **A** Twelve labial gland samples used in this study. **B** The TEM images of the mitochondria in epithelial cells shown at 110,000 × magnification. Scale bar = 500 nm. **C** Expression level of ROS in epithelial cells of labial gland. 4′,6-diamidino-2-phenylindole (DAPI) indicating nucleus (blue light), CY3 indicating ROS (red light). **D** Fe^2+^ level in labial gland tissue **E** Total Fe level in labial gland tissue

In order to further verify the relationship between ferroptosis and local lesions of exocrine gland epithelial cells in pSS patients, we detected several indicators related to ferroptosis in the lip gland tissue of patients. The results showed that there was no significant difference in the size of the labial gland used in this experiment between negative and positive labial gland biopsy (Fig. 7A), but several indicators related to ferroptosis changed significantly. The epithelial cells with positive labial gland biopsy (LGB) had smaller mitochondria, thicker mitochondrial membrane and reduced mitochondrial cristae (Fig. 7B), and contain more reactive oxygen species (Fig. 7C). The Ferrum (Fe) test results showed that the samples with positive LBP had more Fe^2+^ than those with negative LBP (Fig. 7D), but there was no significant change in total Fe level between the two samples (Fig. 7E). These results suggest that there is ferroptosis in the epithelial cells of the labial gland in pSS patients, but there is a significant difference in ferroptosis between the LGB positive and the negative specimens.

### Ferroptosis may be involved in the pathogenesis of endothelial cell disease

**Fig. 8:**
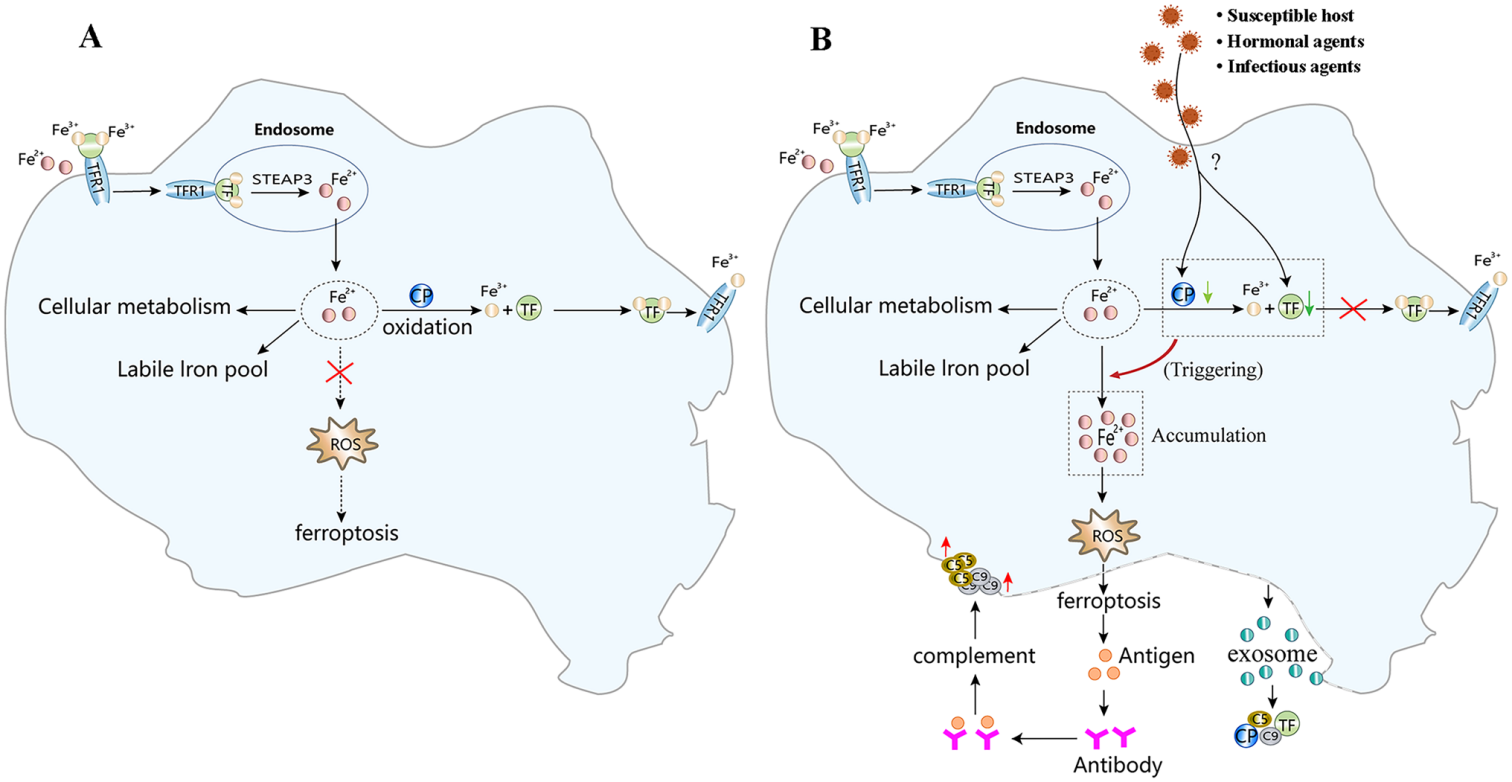
The ferroptosis-related proteins identified via proteomics are involved in the possible mechanism of epithelial cell destruction. **A** A schematic diagram of normal iron metabolism in cells. Intracellular Fe^2+^ is mainly used for cell metabolism and iron storage, while excess iron can be oxidized into Fe^3+^ via CP, which does not induce ferroptosis after being excluded by TF. **B** A schematic diagram of the possible ferroptosis mechanism in epithelial cells. The decreased expression of CP and TF in the pSS epithelial cells may be caused by viral infection and estrogen factors in genetically susceptible hosts. A decrease in CP and TF expression can inhibit the oxidation of Fe^2+^ to Fe^3+^ and iron transport, resulting in Fe^2+^ accumulation and increased ROS, triggering ferroptosis. Epithelial cell ferroptosis can encourage B cells to produce antibodies by releasing autoantigens. This activates the complementary system to form C5 and C9 membrane attack complexes on the epithelial cell membrane, accelerating the destructive epithelial cell lesions. In addition, the exosomes released by the ferroptosis epithelial cells into the blood contain a large number of information molecules that can reflect cell lesions, such as C5, C9, TF, and CP

Combined with the theory of ferroptosis and the existing research results of pSS pathogenesis, a schematic diagram was created of the possible involvement of ferroptosis in the pathogenesis of epithelial cell lesions. Intracellular Fe^2+^ is mainly used for cell metabolism and iron storage, while excess iron can be oxidized into Fe^3+^ via CP, which does not induce ferroptosis (Fig. 8A). It is believed that the CP and TF expression levels in the epithelial cells of genetically susceptible hosts may be significantly reduced when exposed to the joint action of virus infection and hormone factors [26, 27]. As shown in Fig. 8B, this inhibits Fe^2+^ oxidation to Fe^3+^ and its transport, resulting in significant Fe^2+^ accumulation, triggering lipid peroxidation and the accompanying increase in ROS, consequently inducing ferroptosis. Moreover, epithelial cell ferroptosis can release autoantigens to encourage B cells to produce antibodies and activate the complementary system. This causes substantial C5 and C9 accumulation on the epithelial cell membrane to form a membrane attack complex, accelerating the destructive epithelial cell lesions. In addition, epithelial cells can release exosomes during ferroptosis that can reflect cell lesions in the blood, providing a basis for studying plasma exosomes from an epithelial cell injury mechanism standpoint.

## Discussion

The cause of pSS, a chronic autoimmune disease, remains unknown. Epithelial cells are considered crucial in the progression of the disease, as their lesions are not only capable of recruiting mononuclear cells to cause recurrent chronic inflammatory responses and subsequent dry symptoms [28, 29], but also promote multi-system and multi-tissue injury in severe cases [5, 29]. However, the mechanism underlying epithelial cell lesions are not fully understood, and there is a lack of drugs targeting epithelial cells to treat pSS. Since the exosomes in plasma contain information molecules that can reflect the pathological changes in the originating cells, they are attracting increasing attention with regard to the pathogenesis of many diseases [13]. This study aimed to determine whether the DEPs in the plasma exosomes were involved in the cell death pathways, providing an experimental basis for elucidating the mechanism behind the epithelial cell lesions in pSS.

The results show that pSS plasma exosomes contained 24 DEPs, 17 of which displayed significant upregulation, and 7 of which exhibited downregulation. GO enrichment, KEGG, and Reactome pathway analysis showed that these DEPs were closely associated with ferroptosis. It has been reported that salivary gland epithelial cells can release multiple exosomes and contain autoantigens closely related to autoimmune diseases [30]. To verify whether abnormal expression of plasma exosomes involved in ferroptosis is associated with exocrine gland epithelial cell damage, the epithelial cell markers SSA and SSB in the plasma and saliva exosomes were detected. The results showed that the content of these proteins in plasma exosomes of patients was significantly higher than those of healthy controls. Similar results were shown in saliva exosomes, where the content of SSA and SSB in patients was significantly higher than in healthy controls. The abnormal expression of these ferroptosis-related proteins may be related to the damage of the salivary gland epithelial cells. Studies over the years have shown that the main pathological features of the PSS exocrine glands are apoptosis and lymphocyte infiltration [6]. Recently, some studies have shown that apoptosis is often accompanied by ferroptosis [31], apoptosis and necrosis inducers can trigger ferroptosis [32], and T lymphocytes and macrophages also accelerate the occurrence of ferroptosis [33, 34]. It also suggests that plasma exosomal proteins involved in ferroptosis may be related to exosomes released from exocrine gland epithelial cells with localized lesions. Ferroptosis represents a new manner of cell death, which differs from apoptosis. It denotes a process in which the damage caused by iron-dependent lipid peroxidation leads to programmed cell death [35]. It selectively kills target cells, causes tissue damage, activates the immune system by releasing inflammation-related injury molecules and is involved in various signaling pathway-mediated inflammatory responses [10, 36]. These properties indicate that the damage to the exocrine gland of pSS patients and the accompanying inflammatory response is due to ferroptosis, which may be related to the destruction of SS exocrine gland tissue. To further examine the relationship between ferroptosis and epithelial cell injury of pSS exocrine gland, this study took the labial gland as the study object and found that there was ferroptosis in the epithelial cells of the labial gland, and epithelial cells with positive LGB were more susceptible to ferroptosis than those with negative LGB. These findings suggest that there is ferroptosis in the epithelial cells of exocrine glands in pSS patients.

CP and TF represent the downregulated DEPs involved in ferroptosis. CP displays oxidase activity and can oxidize Fe^2+^ into Fe^3+^, while TF is a Fe^3+^ transporter. They play an important role in metabolic iron balance. Downregulated or dysfunctional expression causes an imbalance in the iron homeostasis in cells, increases the Fe^2+^ in cells and increases the production of toxic reactive oxygen species (ROS) substances mediated by iron ions, consequently inducing ferroptosis [10, 36]. This study shows significantly reduced CP and TF expression levels, which may be an important cause of ferroptosis in epithelial cells. However, the specific mechanism underlying ferroptosis remains unclear, while many of the molecules involved have yet to be revealed [37]. Considering the CP and TF involved in ferroptosis, the PPI database was used to determine their interaction with other DEPs. The results showed a clustering network with TF and CP, while direct interaction was evident with the C5, C9, and SERPING1 proteins, representing vital components of the complement membrane attack complex with cells lysis. The cell lysis products of ferroptosis contain many antigens, which can induce the production of antibodies for complementary activation [38]. None of these molecules are reportedly involved in ferroptosis. However, the results of this study indicated that further studies could help definitively clarify this issue. Although these proteins may be involved in ferroptosis, no new proteins have been revealed.

To further understand the role of these proteins during ferroptosis in pSS patients, their expression levels in exosomes and plasma were further examined using ELISA, showing consistency with the proteomic pSS results. The CP and TF expression levels decreased significantly compared to the HCs, while that of C5, C9, and SERPING1 increased. Moreover, these exosomal protein expression levels in sSS and RA were similar to those in pSS. As the sSS collected in this study contains not only exocrine epithelial cell damage, but also cartilage cell damage from RA disease [39], it suggests that exosomal proteins in plasma involved in ferroptosis may also be related to the destruction of chondrocytes. It is worth mentioning that the expression levels of these proteins are notably different in the plasma than in the exosomes. No substantial variation was evident in the CP, TF, HP, and SERPING1 expression in pSS, sSS, RA, and nSS, compared with the HC participants. These results suggest that the molecular protein content may change significantly in exosomes in the absence of systemic circulation modification, further indicating that exosome proteins are more conducive to studying disease markers and pathogenesis. Similarly, compared with the HCs, the C5 expression was substantially lower in sSS, and the C9 expression decreased significantly in pSS, sSS, and RA. These results suggest that a decrease in the complement C5 and C9 content in systemic circulation increases the C5 and C9 accumulated on the cell membrane in the local lesions, enhancing the cytolytic effect of the complement in the local lesions and increasing the content of complement C5 and C9 in the exosomes released into the blood. These findings provide evidence for the comparative study of the expression differences between exosomal proteins and plasma proteins, as well as the examination of exosomal proteins during the pathogenesis and as markers of diseases.

In summary, the study showed that pSS plasma-derived exosomes contained DEPs associated with ferroptosis. Although the study results provide a fresh point of view for revealing the underlying mechanism of epithelial cell damage, the role of ferroptosis in the destruction of epithelial cells must be confirmed via further investigation.

### Supplementary Information

Below is the link to the electronic supplementary material.

Supplementary file1 (DOCX 12 KB)

Supplementary file2 (DOCX 12 KB)

Supplementary file3 (DOCX 14 KB)

Supplementary file4 (DOCX 17 KB)

Supplementary file5 (DOCX 17 KB)

Supplementary file6 (DOCX 17 KB)

## Data Availability

The original contributions presented in the study are included in the article supplementary material, further inquiries can be directed to the corresponding authors.
