# Supplementary material for: The plasma exosomes from patients with primary Sjögren’s syndrome contain epithelial cell–derived proteins involved in ferroptosis
Source: J Mol Med (Berl). 2023 Sep 1;101(10):1289–304. doi: 10.1007/s00109-023-02361-0 (PMC10560162; doi:10.1007/s00109-023-02361-0)
Supplement: Supplementary file 1 — Supplementary file1 (DOCX 12 KB) [file 109_2023_2361_MOESM1_ESM.docx]

**Table 1** Clinical characteristics of patients with pSS, sSS, RA and nSS

| Parameter | pSS(n=86) | sSS(n=18) | RA(n=46) | nSS(n=16) |
| --- | --- | --- | --- | --- |
| Disease duration(year) | 4(2-6) | 6(4-10) | 7(5-11) | 3(2-4) |
| Duration of dryness (year) | 4(2-6) | 3(2-4) | - | 3(2-4) |
| Schirmer’s I test positive,n(%) | 86 (100.00) | 18 (100.00) | - | 8 (50.00) |
| Unstimulated salivary  flow positive,n(%) | 81 (94.19) | 16 (88.89) | - | 5 (27.78) |
| Focus score positive,n(100%) | 86 (100.00) | 18 (100.00) | - | 0 (0.00) |
| RF positive, n (%) | 26 (30.23) | 15 (83.33) | 37 (80.43) | 0 (0.0) |
| ACCP positive, n (%) | 0 (0.00) | 8 (44.44) | 23 (50.00) | 0 (0.00) |
| Anti-SSA positive , n (%) | 75(87.21) | 12 (66.67) | 0 (0.00) | 0 (0.00) |
| Anti-SSB positive, n (%) | 43 (50.00) | 6(33.33) | 0 (0.00) | 0 (0.00) |
