# Supplementary material for: The plasma exosomes from patients with primary Sjögren’s syndrome contain epithelial cell–derived proteins involved in ferroptosis
Source: J Mol Med (Berl). 2023 Sep 1;101(10):1289–304. doi: 10.1007/s00109-023-02361-0 (PMC10560162; doi:10.1007/s00109-023-02361-0)
Supplement: Supplementary file 2 — Supplementary file2 (DOCX 12 KB) [file 109_2023_2361_MOESM2_ESM.docx]

Table 2 Baseline parameters of patients and healthy controls(HCs).

| Parameter | pSS(n=86) | sSS(n=18) | RA(n=46) | nSS(n=16) | HCs(n=46) |
| --- | --- | --- | --- | --- | --- |
| Sex(females/males) | 26/0 | 8/0 | 7/5 | 6/0 | 8/0 |
| Age (year) | 50.35±9.21 | 52.35±7.35 | 53.73±8.14 | 49.03±8.23 | 47.61±7.81 |
| GLU(mmol/L) | 4.71±0.42 | 5.11±0.72 | 4.66±0.73 | 4.26±0.44 | 4.47±0.24 |
| ESR (mm/h) | 5.65±4.2 | 11.61±7.52* | 12.63±6.12* | 6.43±3.77 | 4.61±2.23 |
| CRP(mg/L) | 5.35±4.33 | 29.78±18.55* | 24.68±15.87* | 5.65±4.2 | 6.65±2.27 |
| Leukocytes (×10^12^/L) | 4.61±0.72 | 5.63±1.05 | 4.81±0.85 | 4.61±0.65 | 4.11±0.54 |
| Platelets (×10^9^/L) | 100.22±21.3 | 99.27±21.73 | 101.71±28.22 | 106.22±21.23 | 120.72±18.33 |
| Urea (mmoL/L) | 4.51 ± 1.12 | 5.54 ± 1.33 | 6.51 ± 2.65 | 3.52 ± 1.08 | 3.51 ± 0.25 |
| TC (mmoL/L) | 5.60 ± 1.12 | 4.72 ± 0.81 | 6.23 ± 2.07 | 6.60 ± 2.15 | 4.62 ± 1.12 |
| LDL (mmoL/L) | 4.36 ± 0.91 | 6.56 ± 2.56 | 6.58 ± 1.44 | 3.77 ± 0.37 | 3.16 ± 0.81 |

* indicate the comparison between the disease group and the healthy group ,P < 0.05 .
