# Supplementary material for: The plasma exosomes from patients with primary Sjögren’s syndrome contain epithelial cell–derived proteins involved in ferroptosis
Source: J Mol Med (Berl). 2023 Sep 1;101(10):1289–304. doi: 10.1007/s00109-023-02361-0 (PMC10560162; doi:10.1007/s00109-023-02361-0)
Supplement: Supplementary file 3 — Supplementary file3 (DOCX 14 KB) [file 109_2023_2361_MOESM3_ESM.docx]

**Supplementary Table 3** GO enrichment (BP) analysis of DEPs from the exosomes of pSS patients and HCs (The top 11terms)

| GO ID | GO term | P value | Enrichment | Enriched proteins |
| --- | --- | --- | --- | --- |
| GO:0007257 | activation of JUN kinase activity | 0.0052 | 2.2861 | ERN1,TF |
| GO:0032868 | response to insulin | 0.0052 | 2.2861 | IGFBP2 |
| GO:0055072 | iron ion homeostasis | 0.0148 | 1.8291 | CP,TF |
| GO:0007165 | signal transduction | 0.0219 | 1.6603 | IGFBP2 |
| GO:0007597 | blood coagulation, intrinsic pathway | 0.0219 | 1.6603 | IGFBP6,SERPIG1 |
| GO:0006879 | cellular iron ion homeostasis | 0.0283 | 1.5481 | F9, VWF,CP,TF |
| GO:0090090 | Wnt signaling pathway | 0.0283 | 1.5481 | IGFBP2,IGFBP6 |
| GO:0006826 | iron ion transport | 0.0283 | 1.5481 | CP,TF,IGFBP2 |
| GO:0043567 | regulation of ILGFR sgnaling pathway | 0.0451 | 1.3462 | IGFBP6 |
| GO:0006957 | alternative pathway | 0.0493 | 1.3067 | CFD,C9,C5 |
| GO:0000187 | activation of MAPK activity | 0.0646 | 1.1899 | IGFBP6,C5 |
