# Supplementary material for: The plasma exosomes from patients with primary Sjögren’s syndrome contain epithelial cell–derived proteins involved in ferroptosis
Source: J Mol Med (Berl). 2023 Sep 1;101(10):1289–304. doi: 10.1007/s00109-023-02361-0 (PMC10560162; doi:10.1007/s00109-023-02361-0)
Supplement: Supplementary file 4 — Supplementary file4 (DOCX 17 KB) [file 109_2023_2361_MOESM4_ESM.docx]

**SupplementaryTable2** GO enrichment (CC) analysis of DEPs from the exosomes of pSS patients and HCs (The top 11terms)

| GO ID | GO term | P value | Enrichment | Enriched proteins |
| --- | --- | --- | --- | --- |
| GO:0016324 | apical plasma membrane | 0.0154 | 1.8124 | IGFBP2,TF |
| GO:0005770 | late endosome | 0.0154 | 1.8124 | TF,PSAP |
| GO:0031410 | cytoplasmic vesicle | 0.0467 | 1.3304 | IGFBP2,TF |
| GO:0035580 | specific granule lumen | 0.0519 | 1.2848 | HP,CAMP |
| GO:0005765 | lysosomal membrane | 0.0669 | 1.1746 | CP,PSAP |
| GO:1990604 | IRE1-TRAF2-ASK1 complex | 0.0747 | 1.1266 | ERN1 |
| GO:0032839 | dendrite cytoplasm | 0.0747 | 1.1266 | SFPQ |
| GO:0033093 | Weibel-Palade body | 0.0747 | 1.1266 | VWF |
| GO:1990597 | AIP1-IRE1 complex | 0.0747 | 1.1266 | ERN1 |
| GO:0009925 | basal plasma membrane | 0.0747 | 1.1266 | TF |
| GO:1990332 | Ire1 complex | 0.0747 | 1.1266 | ERN1 |
