# Supplementary material for: The plasma exosomes from patients with primary Sjögren’s syndrome contain epithelial cell–derived proteins involved in ferroptosis
Source: J Mol Med (Berl). 2023 Sep 1;101(10):1289–304. doi: 10.1007/s00109-023-02361-0 (PMC10560162; doi:10.1007/s00109-023-02361-0)
Supplement: Supplementary file 5 — Supplementary file5 (DOCX 17 KB) [file 109_2023_2361_MOESM5_ESM.docx]

**Supplementary Table3** KEEG enrichment analysis of DEPs from the exosomes of pSS patients and HCs (The top 13terms)

| KEEG ID | KEEG term | Pvalue | Enrichment | Enriched proteins |
| --- | --- | --- | --- | --- |
| path:hsa04216 | Ferroptosis | 0.0291 | 1.5358 | CP,TF |
| path:hsa00860 | Porphyrin and chlorophyll metabolism | 0.1049 | 0.9792 | CP |
| path:hsa04978 | Mineral absorption | 0.1049 | 0.9792 | TF |
| path:hsa05017 | Spinocerebellar ataxia | 0.1049 | 0.9792 | ERN1 |
| path:hsa04142 | Lysosome | 0.1049 | 0.9792 | PSAP |
| path:hsa05150 | Staphylococcus aureus infection | 0.1087 | 0.9639 | CFD,KRT10 |
| path:hsa04140 | Autophagy - animal | 0.1994 | 0.7002 | MASP2,C5  ERN1 |
| path:hsa04932 | Non-alcoholic fatty liver disease | 0.1994 | 0.7002 | ERN1 |
| path:hsa04621 | NOD-like receptor signaling pathway | 0.1994 | 0.7002 | CAMP |
| path:hsa04210 | Apoptosis | 0.1994 | 0.7002 | ERN1 |
| path:hsa04970 | Salivary secretion | 0.2846 | 0.5457 | CAMP |
| path:hsa04141 | Protein process in endoplasm reticulum | 0.2846 | 0.5457 | ERN1 |
| path:hsa05010 | Alzheimer disease | 0.2846 | 0.5457 | ERN1 |
