# Supplementary material for: The plasma exosomes from patients with primary Sjögren’s syndrome contain epithelial cell–derived proteins involved in ferroptosis
Source: J Mol Med (Berl). 2023 Sep 1;101(10):1289–304. doi: 10.1007/s00109-023-02361-0 (PMC10560162; doi:10.1007/s00109-023-02361-0)
Supplement: Supplementary file 6 — Supplementary file6 (DOCX 17 KB) [file 109_2023_2361_MOESM6_ESM.docx]

**Supplementary Table4** Reactome enrichment analysis of DEPs from the exosomes of pSS patients and HCs (The top 13 terms)

| Reactone ID | Reactone term | Pvalue | | | Enrichment | | Enriched proteins | |
| --- | --- | --- | --- | --- | --- | --- | --- | --- |
| R-HSA-917937 | Iron uptake and transport | | 0.0056 | | | 2.2518 | | CP,TF |
| R-HSA-375276 | Peptide ligand-binding receptors | | | 0.0695 | | 1.1577 | | C5,PSAP |
| R-HSA-5619049 | Causing hemochromatosis 4 | | | 0.0766 | | 1.1156 | | CP |
| R-HSA-430116 | GP1b-IX-V activation signalling | | | 0.0766 | | 1.1156 | | VWF |
| R-HSA-8849468 | PTK6 Involved in RNA Processing | | | 0.0766 | | 1.1156 | | SFPQ |
| R-HSA-425410 | Metal ion SLC transporters | | | 0.0766 | | 1.1156 | | CP |
| R-HSA-9673240 | Defective gamma-carboxylation of F9 | | | 0.0766 | | 1.1156 | | F9 |
| R-HSA-9672393 | Defective F8 binding to VWF | | | 0.0766 | | 1.1156 | | VWF |
| R-HSA-9635465 | Suppression of apoptosis | | | 0.0766 | | 1.1156 | | SFPQ |
| R-HSA-1660662 | Glycosphingolipid metabolism | | | 0.0766 | | 1.1156 | | PSAP |
| R-HSA-75892 | Platelet Adhesion to exposed collagen | | | 0.0766 | | 1.1156 | | VWF |
| R-HSA-9673218 | Defective F9 secretion | | | 0.0766 | | 1.1156 | | F9 |
| R-HSA-5619060 | Defective CP causes aceruloplasminemia | | | 0.0766 | | 1.1156 | | CP |
